# Granulocyte Colony-Stimulating Factor Use in a Large Iranian Hospital: Comparison with American Society of Clinical Oncology (ASCO) Clinical Practice Guideline

**Published:** 2016-04-01

**Authors:** Sarah Mousavi, Mina Dadpoor, Farzaneh Ashrafi

**Affiliations:** 1 Department of Clinical Pharmacy and Pharmacy Practice, Faculty of Pharmacy and Pharmaceutical Sciences, Isfahan University of Medical Sciences, Isfahan, Iran; 2 Pharm D Student, Faculty of Pharmacy and Pharmaceutical Sciences, Isfahan University of Medical Sciences, Isfahan, Iran; 3 Hematology and Medical Oncology Department, School of Medicine, Isfahan University of Medical Sciences, Isfahan, Iran; 4 Acquired Immunodeﬁciency Research Center, Isfahan University of Medical Sciences, Isfahan, Iran

**Keywords:** Granulocyte colony stimulating factor, Clinical audit, Drug utilization review

## Abstract

**Background:** Granulocyte Colony Stimulating Factors (GCSF) is high-cost agents commonly recommended for primary and secondary prophylaxis of chemotherapy-induced neutropenia and febrile neutropenia. GCSFs have been shown to be beneficial in some patient subgroups, although they are probably overused in clinical settings. The American Society of Clinical Oncology (ASCO) guidelines summarize current data on the appropriate use of CSFs. The aim of this study was to assess and audit the use of GCSF in a tertiary care center according to the recommendation of ASCO guideline.

**Subjects and Methods:** A prospective observational study from November 2014 to June 2015 was performed on all patients prescribed with filgrastim in the large teaching hospital (Isfahan, Iran). Data was collected on demographics, indication, dosing regimen and duration of treatment, the Absolute Neutrophil Count (ANC) and patient outcome.

**Results:** 91 patients were recorded over the period of the study. 63.7% of prescription complied with the ASCO guideline. Febrile neutropenia post chemotherapy/radiotherapy was the most common appropriate indication (29.3%) followed by primary prophylaxis (25.8%). Fourteen (32%) patients showed ANC recovery in 1-3 days and 16 (37%) within 4-7 days. Ten patients (23%) showed no recovery. The overall mortality was 8 (8.8%) patients.

**Conclusion:** This study revealed that at least one-third of prescribed GCSF was not in accordance with ASCO guideline. Considering the high cost of GCSF in our country and limitation of our resources, we proposed cost-effectiveness studies on GCSF treatment and also the development of a national guideline for optimizing GCSF use.

## Introduction

 Granulocyte (GCSF) and granulocyte-macrophage colony stimulating factor (GM-CSF) are hematopoietic hormones promoting the growth and maturation of myeloid cells and in particular proliferation and differentiation of neutrophils.^   1 ^ The pharmaceutical analogs of naturally occurring GCSF are called filgrastim and lenograstim. These factors are in clinical use in various clinical situations, including treatment of neutropenia, chemotherapy-induced myelosuppression, recovering from aplasia after allogeneic or autologous bone marrow transplantation, congenital or cyclical neutropenia. 2^,^^3^  Clinical trials have indicated that the CSFs enhanced patient quality of life^   4 ^ and reduced hospital costs^   5 ^ by reducing the days of hospitalization and total number of days of treatment with parenteral antibiotics. 6^,^^7^ 

Several society and networks developed clinical guidelines to optimize GCSF use. Such as the American Society of Clinical Oncology (ASCO),^   8 ^ the National Comprehensive Cancer Network (NCCN),^   9 ^ and the European Organization for Research and Treatment of Cancer (EORTC).^   2 ^ The ASCO adopted evidence-based guidelines in 1994 and then updated the guideline in 1996, 1997, 2000 and 2006. Lists of the relevant updated recommendations for the use of GCSF are as follows:^   8 ^

1. Primary administration of GCSF should be reserved for patients expected to experience a 20% or greater risk of febrile neutropenia based on age, medical history, disease characteristics and risk of myelotoxicity associated with a chemotherapy regimen.

2. Secondary prophylactic GCSF administration can be considered in patients who have previously experienced an episode of febrile neutropenia when a reduction in dose of chemotherapy is not appropriate.

3. GCSF can be considered as adjunctive treatment of febrile neutropenia for patients at high risk for infection complications (e.g. patients with pneumonia, hypotension, sepsis syndrome, multiorgan dysfunction, fungal infection, uncontrolled primary disease, or profound neutropenia (absolute neutrophil count (ANC) < 100 μL).

4. As adjunct to peripheral blood progenitor cell (PBPC) mobilization and post-transplantation, GCSF are effective.

5. In patients with Acute Myeloblastic Leukemia (AML), GCSF use is recommended with initial or repeat induction chemotherapy or completion consolidation therapy.

6. Also GCSF recommended following completion of initial induction therapy or first post-remission course of chemotherapy following Acute Lymphoblastic Leukemia (ALL).

7. As prophylaxis in patients 65 years and older with diffuse aggressive lymphoma underlying CHOP or more aggressive regimens.

8. For increase of ANC in patients with myelodysplastic syndrome or aplastic anemia.

The aim of this study was to provide data on the pattern of use of GCSF in a tertiary care teaching hospital and to assess the extent of compliance with the ASCO guideline.

## SUBJECTS AND METHODS

 This was a prospective observational study which conducted in an 850 bed university hospital with inpatient and outpatient care services, affiliated to Isfahan University of Medical Sciences, in Isfahan, Iran. All patients who received GCSF from November 2014 to June 2015 were identified and selected through the pharmacy computer system. The charts of each patient were reviewed and data were retrieved. All included patients followed-up till discharge or death.

A data collection standard form was developed, pretested and modified prior to including following data: patient demographic details (ID number, gender, age, weight, etc.), admitting diagnosis, units of admission, dates of admission and discharge, prescribing data for the use of GCSF (including indication, dose, dosing interval, route of administration, duration of therapy, major side effects), types of cancer and chemotherapy regimen, laboratory data (including WBC counts with differential counts, hemoglobin, platelet counts and RBC) and outcomes of GCSF treatment (recovery and non-recovery) and patients (dead or alive). Absolute neutrophil count (ANC) was calculated for each patient [ANC= WBC × total neutrophils (segmented neutrophil % + segmented bands %) ×10].

Drug use was evaluated for appropriateness based on whether ASCO guidelines were adopted. Recovery was considered when ANC rose above 1500 cells/mm^3^ with GCSF treatment. Arbitrary cut off was created based on the presentation of data for ANC, as less than 100 cells/mm^3^, between 100-500 cells/mm^3^, between 500-1000 cells/mm^3^, between 1000-1500 cells/mm^3^ and more than 1500 cells/mm^3^.

Data are summarized as relative frequencies for categorical variables and mean (SD) for normally distributed continuous variables. Calculations were made with SPSS 20.0, Chicago, USA.

## Results

 A total of 91 patients received GCSF during the study period. The mean age was 42.7 ± 17.2 (range 16-80) years. Male subjects constituted 57.1% (N= 52) of patients. There were 34 patients with hematological malignancy and 21 patients with solid tumors. Table 1 shows the baseline demographics, types of cancer and WBC and ANC of patients at presentation and at GCSF initiation.

In 63.7% of the cases (58/91), GCSF was prescribed according to the audit criterion of ASCO guideline. Tables 2 and 3 show the indication of GCSF administration. Among the appropriate indication, the majority of patients (17/58, 29.3%) were received GCSF for febrile neutropenia post chemotherapy, followed by primary prophylaxis in 15 (25.8%) cases. In 13 treatments, primary prophylaxis (N=15) were prescribed because febrile neutropenia (FN) risk associated with chemotherapy regimen was ≥ 20%. In 2 remaining cases, GCSF was prescribed in one case with diffuse aggressive lymphoma and age >65 years, and another patient was high risk for infection complication.

As shown in Table 1, 72 patients had ANC more than 1000 cells/mm^3 ^at presentation, whereas the median of ANC at GCSF initiation was > 1500 cells/mm^3^ in 57 cases.

16 out of 43 courses of GCSF treatment (include FN + secondary prophylaxis cases), showed an ANC recovery in 4-7 days (37.2%) while ten patients had no recovery. Fourteen (32.5%) had ANC recovery in 1-3 days and three patients had recovered in more than 7 days. The overall mortality was 8 patients (8.8%). Seven of patients with unjustified indications for GCSF had ANC recovery, while five of them died (5/33, 15.2%). The ANC has been recovered in 14 patients out of 58 justified GCSF courses.

**Table 1:** Patient characteristics

**Characteristics**	**Number of patients (N=91)**
Age, years (range)	Mean: 42.7 ± 17.2 (16-80) Median: 40
Sex, Male/Female	52/39 (57.1%/42.9%)
**Types of cancer**	
***Hematological malignancies***	
a. Lymphoma	10
b. Leukemia ALL AML Others	12 8 4
***Solid tumors***	
a. Breast cancer	5
b. Lung cancer	3
c. Gastrointestinal cancer	6
d. Choriocarcinoma	3
e. Sarcoma	2
f. Prostate/ovarian cancer	2
**Median ANC at presentation**	
ANC < 100 cells/mm^3^	6
ANC 100-500 cells/mm^3^	7
ANC 500-1000 cells/mm^3^	6
ANC > 1500 cells/mm^3^	72
**Median ANC at GCSF initiation**	
ANC < 100 cells/mm^3^	7
ANC 100-500 cells/mm^3^	20
ANC 500-1000 cells/mm^3^	4
ANC 1000-1500 cells/mm^3^	3
ANC > 1500 cells/mm^3^	57
**Days to ANC recovery**	
No recovery	10 (23%)
1-3 days	14 (32%)
4-7 days	16 (37%)
> 7 days	3 (7%)

**ALL:** Acute Lymphoblastic Leukemia, **AML:** Acute Myeloblastic Leukemia, **GCSF:** Granulocyte Colony Stimulating factor, **ANC:** Absolute Neutrophil Count

All patients received GCSF as a subcutaneous injection. The usual prescribed dose was 5 mcg/kg/day with a median of 300 mcg (according to vial size) and a mean of 373 ± 154 mcg. In 68 patients (74.4%), the total daily dose of GCSF was given four times daily.

**Table 2:** Indication of GCSF courses according to ASCO guideline

**Indications**	**No. of Patients ** **(%) (total N=58)**
Primary prophylaxis	15 (25.8%)
Secondary prophylaxis	8 (13.7%)
Established febrile neutropenia post chemotherapy	17 (29.3%)
Post induction chemotherapy in acute lymphoblastic leukemia	7 (12%)
Post consolidation chemotherapy in acute myeloblastic leukemia	5 (8.6%)
Aplastic anemia/ myelodysplastic syndrome	3/3 (10.3%)

**ASCO:** American Society of Clinical Oncologist, **GCSF:** Granulocyte Colony Stimulating factor

**Table 3:** Inappropriate indications of GCSF which was not compatible with ASCO guideline

**Indications**	**No. of Patients (%) (total N=33)**
Febrile Neutropenia	12 (36.3%)
Kidney/liver transplantation	3/7 (30.3%)
Drug-induced neutropenia	6 (18%)
Sepsis syndrome	3 (9%)
Neutropenia of premature birth	2 (6%)

**ASCO:** American Society of Clinical Oncologist, **GCSF:** Granulocyte Colony Stimulating factor

The remaining (N=23, 25.3%) received twice daily dosing. The mean duration of treatment was 5.3 ± 3.5 days (range 1-20 days). In patients who received chemotherapy regimen, GCSF courses were initiated 24 hours after the last dose of chemotherapy in 7 cases. The rest (N=6) started 72 hours, post chemotherapy and one patient was given GCSF 24 hours before starting chemotherapy cycle because of neutropenia upon admission.

27 out of 91 GCSF courses were prescribed by hematology/oncology specialists (29.7%). The rest was from different specialty (N=64, 70.3%), which among them resident of internal medicine had the highest percentage (31/91, 34%), followed by infectious disease specialist (7/91, 7.7%).

We detected adverse effects in 21 patients (23.1%). Bone pain (N=13, 62%) and runny eyes and nose (N=12, 57.1%) was the most reported adverse effects in patients. In one patient, GCSF use was associated with severe chest pain and headache.

## Discussion

 Our study showed that at least one third of GCSF use in our hospital was not complied with ASCO guideline. A few studies have evaluated the use of GCSF; the number is even smaller for audits of compliance with clinical guidelines. Velasco et al.^   10 ^ evaluated GCSF use in a general hospital in England. A total of 104 GCSF treatments were assessed in this study. The overall compliance with the audit criteria (the ASCO guideline and the hospital guideline) was 72.1%. Among them stem cell transplantation had the most compliance with audit criteria (93.3%) and secondary prophylaxis had the least compliance (6.7%). We do not have bone marrow or stem cell transplantation in our center. So GCSF treatment for febrile neutropenia post chemotherapy was the most indication (17/91, 29.3%). Our hospital is a general referral center and most patients usually admit after first diagnosis or several courses of chemotherapy/radiotherapy. Considering this point, use of GCSF for febrile neutropenia seems logical to be the most common indication.

In this audit, the most prevalent type of malignancy was ALL (12/91, 13.2%), which at least seven (21%) of them received GCSF for post induction chemotherapy. Among the solid tumors, the most common type was gastrointestinal (GI) cancer (N=6) followed by breast cancer (N=5). All of these patients received GCSF for treatment of febrile neutropenia. Based on Iranian cancer registry data,^   11 ^ 2009-10, the most common cancer among women was breast cancer (23%) followed by skin (non-melanoma) (10.1%); and in men’s skin(non-melanoma) (14%) and stomach cancer (11.9%) were among the most prevalent cancer, respectively. However the incidence rate of GI cancers is increasing in Iran.^   12 ^ Fourteen patients undergo chemotherapy for solid tumors during our study period. DCF (Docetaxel + cisplatin + floururacil) and RCHOP (Rituximab, cyclophosphamide, doxorubicin, vincristine, and prednisolone) was among the most commonly used chemotherapy regimens which are associated with FN risk ≥ 20%, which have indicated for primary prophylaxis with GCSF.^   13 ^ Among the 15 courses of GCSF for primary prophylaxis, 13 of them were for prophylaxis in patients receiving chemotherapy regimens with FN risk ≥ 20%. In addition the use of GCSF in post induction chemotherapy in ALL (N=7) and post consolidation chemotherapy in AML (N=5), recommended by the updated 2006 evidence based ASCO guideline.

Approximately, 30% of our GCSF treatment courses were for established febrile neutropenia post chemotherapy. As much as 80% of these patients received GCSF prophylaxis post-chemotherapy, which, may be questioned the efficacy of GCSF as prophylactic regimen. Another explanation is that most patients usually receive a fixed dose regimen of 300 mcg/daily instead of exact weight-based dose of 5 mcg/kg/day.

The majority of our patients (N=22, 66.6%) received GCSF treatment for febrile neutropenia due to other causes except cancer, such as severe infection, kidney and liver transplantation and drug-induced neutropenia. Neither the ASCO nor the IDSA (infectious disease society of America)^14^ recommends GCSF for treatment of established FN or as adjunct to antibacterial therapy. Given the cost, adverse effect and lack of significant clinical benefit, the addition of GCSF in fever and neutropenia cases is generally not advocated. This implied an abuse of GCSF in some reviewed cases of FN. The ASCO strongly recommend against the use of GCSF in FN.

GCSF administered in nine patients in our study, because of drug-induced neutropenia. According to a systematic review by Anderson et al.^   15 ^ in 2007, use of GCSF could reduce the duration of neutropenia (8 days vs. 9 days, P: 0.015) and reduce the proportion of infectious or fatal complications (14% vs. 29%, P: 0.03) in patients receiving GCSF. Therefore, the use of GCSFs for febrile or afebrile neutropenia is not justified according to guidelines, but overuse of them in such cases attributed to physician concerns about the outcome of patients.

The median of ANC at GCSF initiation was more than 500 cells/mm^3^ in 26 out of 58 cases and 20/33 patients in appropriate and inappropriate groups, respectively.

According to ASCO guideline, GCSF injection should continue until the neutrophil count has recovered to > 1000 cells/mm^3^ on two consecutive days. This will require a minimum of 5 days of treatment. As shown in Table 4, ANC increased in both groups after GCSF administration. ANC parameters and the predicted time to neutrophil recovery following the nadir, usually determine the duration of GCSF therapy. In our patients’ sample, the mean duration of GCSF therapy was 5 days. However, the range was very wide (1-20 days). The reason is that in 37 out of 91 cases, GCSF was used for prolonged febrile neutropenia.

In our study, most of the GCSF administration as primary prophylaxis lasted less than seven days. The majority of patients (32.7%) had ANC recovery in 4-7 days, which is comparable with other studies such as Sheridan et al.^   16 ^ and Carbonero et al.^   17 ^. Most of our patients (N=34) had a hematological malignancy which usually takes longer time to recover compared with solid tumors.^   18 ^ Delayed ANC recovery was associated with longer periods of hospitalization which not only increase the cost, but also adversely affect the outcome. In our study, three (7%) patients took longer than 7 days for ANC to recover and hence had longer hospital stays, which is acceptable.

On the other hand, in ten patients (23.2%) ANC did not recover to >1000 cells/mm^3^. The median of the ANC at presentation, types of malignancy and presence of other comorbidconditions such as infection or hyperglycemia are other factors that determine the response to GCSF. Apart from the effect on ANC recovery, several studies proved that GCSF products reduce morbidity and mortality in patients receiving chemotherapy treatment. ^19^^,^^20^ 

Our results show that in 36.5% of our patients, the use of GCSF is not supported by the ASCO guideline. GCSF was administered in 22 patients who developed febrile neutropenia due to other causes except cancer, such as kidney and liver transplantation (N=10). Winston et al.^   21 ^ evaluate GCSF efficacy in liver transplant patients (administered 100-300 mcg/day GCSF for maximum of 21 days, N=114) and concluded that despite producing a substantial increase in WBC count after transplantation, GCSF had no beneficial effects on infection, rejection or survival of patients compared to placebo (N=58). On the other hand, Foster et al.^   22 ^ Administered 5-10 mcg/kg/day GCSF in 37 liver transplant patients for 7-10 days. They concluded that GCSF-treated patients had lower episode of sepsis per patient (P<0.02) and acute rejection (P<0.01). In our study, seven of ten cases of kidney and liver transplantation had neutropenia and in all of them neutropenia resolved after GCSF initiation.

**Table 4:** Median of ANC at presentation and after GCSF administration in both appropriate and inappropriate indications

**Median of ANC **	**No. of patients**
**Appropriate indication of GCSF(N=58)**	
**Median of ANC at presentation **	
ANC < 100 cells/mm^3^	22
ANC 100-500 cells/mm^3^	5
ANC 500-1000 cells/mm^3^	5
ANC > 1500 cells/mm^3^	27
**Median of ANC at GCSF initiation**	
ANC < 100 cells/mm^3^	25
ANC 100-500 cells/mm^3^	7
ANC 500-1000 cells/mm^3^	6
ANC > 1500 cells/mm^3^	20
**Median of ANC at the end of GCSF treatment**	
ANC < 100 cells/mm^3^	6
ANC 100-500 cells/mm^3^	15
ANC 500-1000 cells/mm^3^	1
ANC > 1500 cells/mm^3^	37
**Inappropriate indication of GCSF(N=33)**	
**Median of ANC at presentation **	
ANC < 100 cells/mm^3^	5
ANC 100-500 cells/mm^3^	2
ANC 500-1000 cells/mm^3^	2
ANC > 1500 cells/mm^3^	24
**Median of ANC at GCSF initiation**	
ANC < 100 cells/mm^3^	5
ANC 100-500 cells/mm^3^	8
ANC 500-1000 cells/mm^3^	8
ANC > 1500 cells/mm^3^	12
**Median of ANC at the end of GCSF treatment**	
ANC < 100 cells/mm^3^	2
ANC 100-500 cells/mm^3^	6
ANC 500-1000 cells/mm^3^	3
ANC > 1500 cells/mm^3^	22

**GCSF:** Granulocyte Colony Stimulating factor, **ANC:** Absolute Neutrophil Count

Recent meta-analysis of Bo et al.^   23 ^ on 12 trials with 2380 septic patients showed that use of GCSF and GM-CSF did not reduce hospital mortality (RR:0.97, 95% CI: 0,69-1.36, P: 0.86); However, GCSF therapy significantly increased the reversal rate from infection (P:0.02). The authors did not recommend routine use of GCSF in patients with sepsis based on current evidence. Three of our patients received GCSF because of sepsis/septic shock. Only in one of them ANC recovered after GCSF initiation and one patient died on the day of GCSF administration because of underlying disease severity. Data from large randomized controlled, well-designed studies are needed to justify GCSF use in patients with sepsis.

## CONCLUSION

 In conclusion, the overall compliance with the audit criteria (ASCO guideline) was 63.7% (58/91) in our study. GCSF is an established effective therapy in approved indication. In our study, use of GCSF accelerates neutrophil recovery in both groups (appropriate or inappropriate according to guideline). Overall mortality was higher in the unjustified indications. Our resources are constrained in our country, so use of high cost medication like GCSF should be rational and optimized. We propose to design and implementing of studies to evaluate the cost-effectiveness of GCSF treatment and development of national guideline for optimizing the use of GCSF treatment.
